# Supplementary material for: Causal Relationship between Aging and Anorexia Nervosa: A White-Matter-Microstructure-Mediated Mendelian Randomization Analysis
Source: Biomedicines. 2024 Aug 16;12(8):1874. doi: 10.3390/biomedicines12081874 (PMC11351342; doi:10.3390/biomedicines12081874)
Supplement: Supplementary file 1 [file biomedicines-12-01874-s001.zip › Supplementary Figure/Figure S4/Singe SNP plot of MR analysis for TL to ubm-b-1894.pdf]

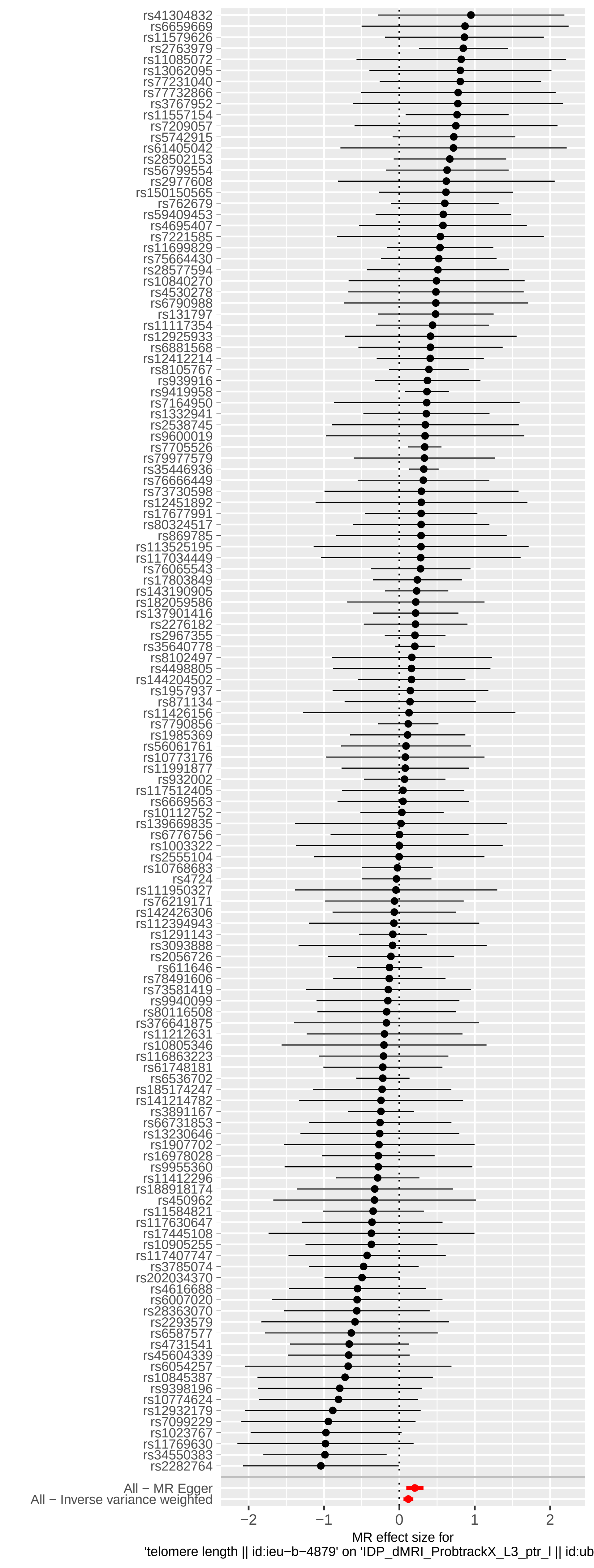
rs41304832
rs6659669
rs11579626
rs2763979
rs11085072
rs13062095
rs77231040
rs77732866
rs3767952
rs11557154
rs7209057
rs5742915
rs61405042
rs28502153
rs56799554
rs2977608
rs150150565
rs762679
rs59409453
rs4695407
rs7221585
rs11699829
rs75664430
rs28577594
rs10840270
rs4530278
rs6790988
rs131797
rs11117354
rs12925933
rs6881568
rs12412214
rs8105767
rs939916
rs9419958
rs7164950
rs1332941
rs2538745
rs9600019
rs7705526
rs79977579
rs35446936
rs76666449
rs73730598
rs12451892
rs17677991
rs80324517
rs869785
rs113525195
rs117034449
rs76065543
rs17803849
rs143190905
rs182059586
rs137901416
rs2276182
rs2967355
rs35640778
rs8102497
rs4498805
rs144204502
rs1957937
rs871134
rs11426156
rs7790856
rs1985369
rs56061761
rs10773176
rs11991877
rs932002
rs117512405
rs6669563
rs10112752
rs139669835
rs6776756
rs1003322
rs2555104
rs10768683
rs4724
rs111950327
rs76219171
rs142426306
rs112394943
rs1291143
rs3093888
rs2056726
rs611646
rs78491606
rs73581419
rs9940099
rs80116508
rs376641875
rs11212631
rs10805346
rs116863223
rs61748181
rs6536702
rs185174247
rs141214782
rs3891167
rs66731853
rs13230646
rs1907702
rs16978028
rs9955360
rs11412296
rs188918174
rs450962
rs11584821
rs117630647
rs17445108
rs10905255
rs117407747
rs3785074
rs202034370
rs4616688
rs6007020
rs28363070
rs2293579
rs6587577
rs4731541
rs45604339
rs6054257
rs10845387
rs9398196
rs10774624
rs12932179
rs7099229
rs1023767
rs11769630
rs34550383
rs2282764
All − MR Egger
All − Inverse variance weighted
−2
−1
0
1
2
MR effect size for
'telomere length || id:ieu−b−4879' on 'IDP_dMRI_ProbtrackX_L3_ptr_l || id:ub
